# Concurrent Androgen Deprivation Therapy for Prostate Cancer Improves Survival for Synchronous or Metachronous Non-Small Cell Lung Cancer: A SEER–Medicare Database Analysis

**DOI:** 10.3390/cancers14133206

**Published:** 2022-06-30

**Authors:** Bassel Nazha, Chao Zhang, Zhengjia Chen, Camille Ragin, Taofeek K. Owonikoko

**Affiliations:** 1 Department of Hematology and Medical Oncology, Winship Cancer Institute of Emory University, Atlanta, GA 30322, USA; 2 Department of Biostatistics, Rollins School of Public Health, Winship Cancer Institute of Emory University, Atlanta, GA 30322, USA; chaozhang73@gmail.com; 3 Division of Epidemiology and Biostatistics, School of Public Health, University of Illinois at Chicago, Chicago, IL 60607, USA; zchen38@emory.edu; 4 Biostatistics Shared Resource Core, University of Illinois Cancer Center, Chicago, IL 60607, USA; 5 Department of Radiation Oncology, Fox Chase Cancer Center, Philadelphia, PA 19111, USA; camille.ragin@fccc.edu; 6 Department of Medicine, Division of Hematology/Oncology, University of Pittsburgh, UPMC Hillman Cancer Center, 5150 Centre Avenue, Pittsburgh, PA 15260, USA; owonikokotk2@upmc.edu

**Keywords:** prostate cancer, lung cancer, hormonal therapy, androgen deprivation therapy, SEER

## Abstract

**Simple Summary:**

The study showed that androgen deprivation therapy for a preceding diagnosis of prostate cancer is associated with prolonged survival among patients who subsequently develop lung cancer. These population-level findings support a role of the androgen receptor in lung cancer.

**Abstract:**

Introduction: The crosstalk between receptor kinase signaling, such as EGFR and androgen receptor signaling, suggests a potential interaction between androgen deprivation therapy (ADT) and lung cancer outcome. Methods: We employed the SEER–Medicare data of lung cancer patients diagnosed between 1988 and 2005 to test for an association between ADT for prostate cancer and lung cancer outcome. We employed the Kaplan–Meier method and Cox proportional hazard with log-rank test model to assess any significant impact of ADT on survival. Results: We included data from 367,750 lung cancer patients; 17.4%, 2.9%, 33.6% and 46.1% with stages I, II, III and IV, respectively; 84.5% were >65 years; 57.2% males; 84.2% Caucasians and 9.3% Blacks. There were 11,061 patients (3%) with an initial prostate cancer diagnosis followed by lung cancer (P-L group); 3017 (0.8%) with an initial diagnosis of lung cancer and subsequent prostate cancer diagnosis (L-P group); the remainder had only lung cancer (L group). Stage I lung cancer was most common in the L-P group compared to the L and P-L groups—54% vs. 17.13% vs. 17.92%, *p* < 0.0001 for L-P, L and P-L, respectively. The median OS for lung cancer diagnosis was 93 months versus 10 and 9 months, respectively, for the L-P, L and P-L subgroups. ADT was associated with improved survival on multivariate analysis, especially in Caucasian patients (HR of death: 0.86; 95% CI: 0.76–0.97; *p* = 0.012). Conclusion: ADT was associated with improved outcome for NSCLC, in line with the hypothesis of a role for the androgen receptor in lung cancer. Our findings support a systematic evaluation of the potential benefit of ADT as a therapy for lung cancer.

## 1. Introduction

Lung cancer is the most common cause of cancer-related mortality in both men and women in the United States, with an estimate of 142,670 deaths in 2019 [1]. Over the last three decades, the incidence of lung cancer steadily decreased among men yet increased among women in the Western world [2], an observation that is partially related to changes in smoking habits but could also be driven by sex hormone differences between men and women. Women have a better overall survival (OS) regardless of stage at presentation compared to men [3]. In addition to gender disparity, with lower overall smoking rates among women [4] and a higher incidence of driver-mutated lung cancer in young non-smoker women [5], differences in endogenous sex hormones between the two sexes have also been implicated as a potential contributor. Estrogen receptor beta (ERbeta) is expressed in non-small cell lung cancer (NSCLC) cell lines as well as in archival tumor specimens, potentially implicating this pathway as a driver of NSCLC growth [6,7]. Previous studies have shown a crosstalk between the estrogen receptor and epidermal growth factor receptor (EGFR) signaling pathway, a known molecular driver of lung cancer [8] (a crosstalk that is conceptually similar to the well-documented one in the immune–angiogenic oncological pathways [9] which are targeted to improve treatment response [10]). Indeed, there have been several prior studies focusing on elucidating the role of estrogen receptor signaling in lung cancer biology and patient survival [11,12,13]. The encouraging results of these early studies led to clinical trials designed to study whether anti-estrogen therapy can potentiate the efficacy of EGFR targeted therapies [14,15]. While the results of these studies suggested a signal of activity, patient selection was suboptimal, as the studies were conducted prior to a full understanding of the necessity for EGFR driver mutation for efficacy of kinase inhibitor therapy.

Despite the high similarity in the sex hormone pathway signaling cascade and the downstream consequences of both androgen and estrogen receptor perturbation by their ligands, the role of androgen receptor expression and signaling in lung cancer pathogenesis remains to be carefully studied.

The exposure of a human lung adenocarcinoma cell line (A549) to androgen significantly altered gene expression, including down-regulation of genes involved in cellular respiration [16]. Androgen deprivation therapy (ADT) is an established hormonal therapy employed to block the synthesis of sex hormones in prostate cancer patients, either as adjuvant intervention or definitive therapy for advanced disease. Retrospective data show that exposure to ADT in patients with lung cancer is associated with better prognosis [17]. In a previous analysis of the Surveillance, Epidemiology, and End Results (SEER) database, men with prior diagnosis of prostate cancer had a 23% lower risk of subsequent lung cancer diagnosis compared to the general population [18]. This reduced risk of secondary lung cancer was less pronounced in Black compared with White patients. However, the study did not address the potential role of ADT in this observation of reduced risk of lung cancer. We employed the linked SEER–Medicare database to systematically assess whether ADT impacts the risk of developing lung cancer and lung cancer patient outcome in the real world.

## 2. Materials and Methods

The primary hypothesis of the study was that ADT use will have a beneficial effect on the outcome for prostate cancer patients with secondary lung cancer diagnosis (compared with patients who did not receive ADT) in terms of survival after lung cancer diagnosis. We used data from the linked SEER–Medicare database, including all lung cancer cases diagnosed between 1988 and 2005, for this analysis. The study was conducted following review and approval by the Emory University Institutional Review Board. Briefly, SEER is the national cancer registry that covers 14% of the US (United States) population. The SEER data linked to Medicare claims allowed us to correlate medical prescription patterns, including ADT, with patient outcomes. We have previously described the SEER and the linked SEER–Medicare databases in detail [19,20,21].

For this analysis, lung cancer cases were classified into three categories: patients diagnosed only with lung cancer (L), patients initially diagnosed with prostate cancer followed by second primary lung cancer diagnosis (P-L) and patients with an initial lung cancer diagnosis who later developed prostate cancer (L-P). To ensure a sufficient duration of ADT therapy, cases with synchronous lung and prostate cancer diagnosis (a latency period shorter than 6 months between the two diagnoses) were excluded from the analysis. The baseline demographics, cancer stage and cancer histology (for both lung and prostate cancer) were collected for the overall population and then compared between the three groups (L, P-L and L-P). Median, 12-month, 24-month and 60-month survival rates calculated from the date of lung cancer diagnosis were estimated for the three patient groups (L, P-L L-P). The L group was stratified by males and females, whereas the L-P and P-L groups only included male patients. The Kaplan–Meier method was used to generate survival curves starting from the time of lung cancer diagnosis as the primary outcome. Using the Cox proportional hazards regression model, a multivariate analysis controlling for important prognostic factors including age, stage and race (White vs. Black patients) was performed to compare the OS in the three groups. All OS analyses in this study were calculated from the time of lung cancer diagnosis.

Secondary analyses for racial differences were conducted within the entire population and separately within the P-L and L-P subgroups. We also assessed for an association between preceding cancer treatment and subsequent cancer diagnosis in both the P-L and L-P groups by comparing the time from the original cancer diagnosis to the subsequent cancer (latency period) for lung and prostate cancer and vice versa. More specifically, the impact of ADT on the latency period for lung cancer diagnosis was analyzed in the P-L group. ADT was defined as the use of luteinizing-hormone-releasing hormone (LHRH) agonists or bilateral orchiectomy using the corresponding treatment codes for these interventions.

Statistical significance along with *p*-values for potential associations were assessed using the Log rank test. The method allowed testing for association between latency period and survival from lung cancer diagnosis. Further, lung cancer survival was evaluated within subsets defined by ADT treatment, stage of lung cancer and stage of prostate cancer. Cox proportional hazards models were also employed to estimate the effect of ADT on lung cancer survival by sequence of cancer diagnosis (P-L or L-P), with adjustment for age and stage of prostate cancer. All analysis was conducted using SAS version 9.3, with a significant level of 0.05.

## 3. Results

### 3.1. Study Population

This SEER–Medicare analysis retrieved included data from 367,750 patients who had a diagnosis of lung cancer. Of these, 84.5% were 65 years or older and 57.2% were males. Distribution by race showed Caucasian patients at 84.2%, Black patients constituted 9.3% and Asian patients 2.3%. The Medicare qualifying event was age for 90.6% of the patients, and 88.2% were deceased at the time of data retrieval (Table 1). Most of the patients (96.2%) had isolated lung cancer diagnosis without concurrent diagnosis of prostate cancer diagnosis (L group); 11,061 patients had a prostate cancer diagnosis followed by a second primary lung cancer diagnosis (P-L group) and 3017 patients had an initial diagnosis of lung cancer diagnosis followed by a second diagnosis of prostate cancer (L-P group) (Table 2). In the P-L subgroup, most patients had advanced lung cancer at diagnosis, with Stage IV (43.7%) and Stage III (33.8%) being the most common (Table 2). In the L-P subgroup, Stage I lung cancer was most common (54%).

Comparison of demographics and stage distribution of lung cancer patients in the three groups revealed that the P-L group had older patients at the time of first diagnosis of lung cancer, where 94.8% were 65 years or older compared with 72.9% in the L-P and 84.3% in L groups. Compared to patients with isolated lung cancer diagnosis (L), Black patients were more likely to have concurrent lung and prostate cancer diagnoses and Caucasian patients were less likely, while Asians showed no difference (Table 2). There were a few notable differences in terms of tumor histology. NSCLC was the most common histologic diagnosis in all three categories, but the rate of SCLC subtype was significantly lower in the P-L and L-P subgroups compared to the L subgroup; 2% vs. 1% vs. 3%; *p* < 0.001).

### 3.2. Lung Cancer Outcome

**Figure 1:**
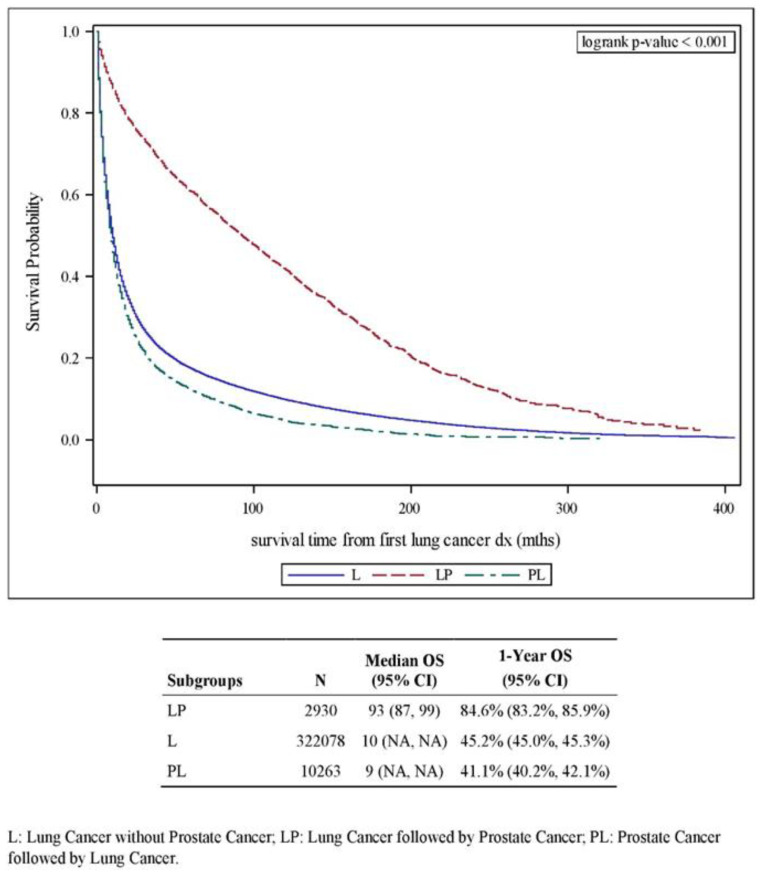
Kaplan–Meier plot for overall survival (OS) comparing the three subgroups, L, P-L and L-P.

The best outcome for lung cancer was observed in the L-P group, with a median OS of 93 months versus 10 and 9 months, respectively, for the L and P-L subgroups (Figure 1) (as expected, since Stage I lung cancer was the most common in the L-P group and Stage IV lung cancer was the most common in the other two groups (Table 2)). On multivariate analysis using female lung cancer patients in the L group as reference and controlling for age, race and stage of lung cancer, the L-P subgroup had the best survival (HR of death: 0.55 (95% CI: 0.52–0.58), *p* < 0.001), whereas the male patient subset in the L and the P-L group had a similar outcome relative to the female patients (HR of death: 1.19 (95% CI: 1.18–1.20) and 1.14 (95% CI: 1.11–1.18), respectively, for L and P-L) (Supplemental Figure S1 and Table 3).

### 3.3. Impact of Concurrent Prostate Cancer Diagnosis on Lung Cancer Diagnosis and Outcome (P-L)

To assess the impact of treatment for the preceding diagnosis of prostate cancer on subsequent lung cancer diagnosis, we compared survival between the P-L, L and L-P subgroups. There was a significant association between stage of prostate cancer and the interval of time to subsequent lung cancer diagnosis. This was noted in the entire population as well as in racial subsets. The longest median latency time of 62 months was observed in patients with Stage III prostate cancer at diagnosis, with a longer latency in White patients at 67 months, versus 44 months in Black patients. In comparison, the median latency period for Stage I prostate cancer patients was 46 months, also with a longer latency in White versus Black patients at 58 months and 42 months, respectively (Table 3). Conversely, there was no difference in the latency period between an initial lung cancer diagnosis and subsequent prostate cancer diagnosis in the L-P subgroup, indicating no clear association between treatment received for different stages of lung cancer and secondary development of prostate cancer (Stage I: 42 m; II: 41 m; III: 48 m; IV: 45 m). Moreover, survival was as expected for the stage of lung cancer and was no different within Black and Caucasian patient subgroups (Supplemental Figure S2).

### 3.4. Androgen Deprivation Therapy and Lung Cancer Outcome

**Figure 2:**
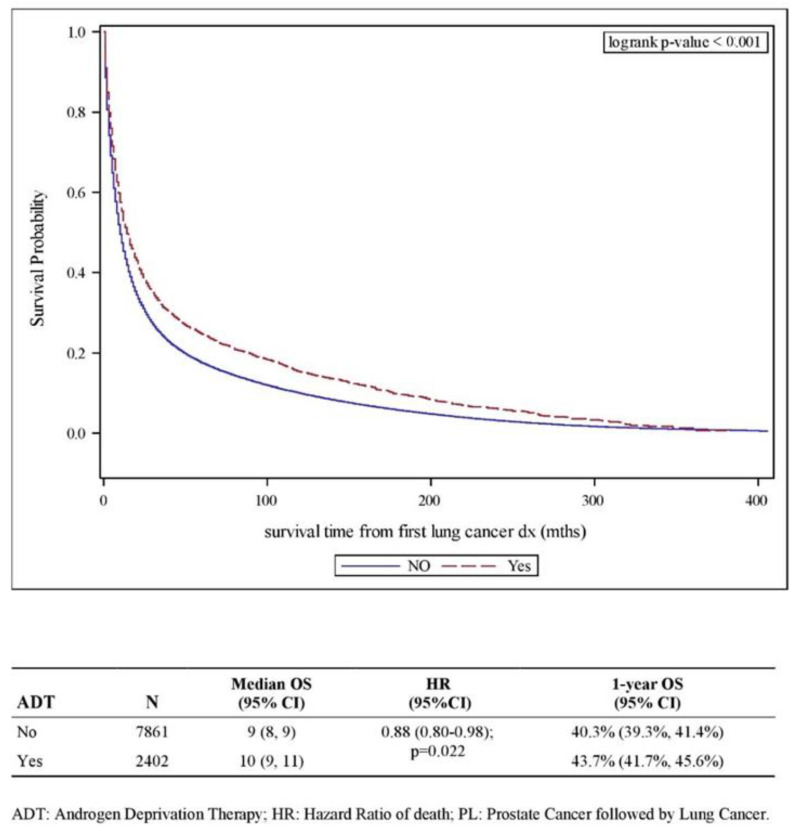
Kaplan–Meier plot for overall survival (OS) in P-L subgroup comparing patients treated with and without ADT.

On univariate analysis, ADT for a preceding diagnosis of prostate cancer was associated with a shorter latency period to the diagnosis of lung cancer (ADT: 40 months vs. no ADT: 47 months, *p* < 0.001) and in both Caucasian patients (41 vs. 48 months, *p* < 0.001), and Black patients (33 vs. 41 months, *p* < 0.001). In addition, ADT for prostate cancer was associated with better survival following lung cancer diagnosis (Figure 2). This benefit was preserved on multivariate analysis while controlling for other factors including age and stage of prostate cancer (HR of death: 0.88; 95% CI: 0.80–0.98, *p* = 0.022). On subset analysis by race, the benefit of ADT was observed in Caucasian patients (HR of death: 0.86; 95% CI: 0.76–0.97; *p* = 0.012) but not in Black patients (HR of death: 0.93; 95% CI: 0.81–1.05; *p* = 0.218) (Supplemental Figure S3).

## 4. Discussion

Despite recent advances with highly effective innovative cancer treatment options, lung cancer remains a deadly disease, with significant unmet need for more effective treatment. Analysis of real-world data is an important source of information that can uncover hitherto unknown associations to guide new treatment options for this disease. Prostate cancer and lung cancer are diseases of the older patient population, with median age at diagnosis of 70 years for lung cancer and 82% of prostate cancer patients being 65 years or older at diagnosis [22]. There are no known shared biological or environmental risk factors for lung and prostate cancer aside from older age. The SEER–Medicare linked database is a well-curated source of diagnosis, treatment and outcome data for US elderly cancer patients [19,23]. This resource enabled us to exploit the management of concurrent diagnosis of lung and prostate cancer in the elderly population to identify potential new strategies for the treatment of lung cancer.

Our analysis showed a significant association between lung cancer outcome and a preceding prostate cancer diagnosis and its treatment. There was the interesting observation that patients diagnosed with Stage III prostate cancer were more likely to have a better outcome with a subsequent diagnosis of lung cancer. We specifically assessed for any impact of ADT on lung cancer, because patients with Stage III prostate cancer were more likely to have received ADT in comparison to Stage I and II patients. Stage III patients were also more likely than Stage IV patients to survive long enough on ADT to develop secondary cancers. Indeed, ADT shortened the latency between prostate cancer and subsequent lung cancer diagnosis. This was observed in both Caucasian and Black patients, although the latency period was shorter in Black patients. There was also an improved outcome for lung cancer in patients exposed to ADT, which, on subset analysis, appeared limited to Caucasian patients. We speculate that this association could be a direct effect of ADT on lung cancer growth and/or the interaction of these treatment agents with standard treatment options for lung cancer.

Various lines of evidence implicate androgen receptor signaling to lung cancer biology. The proposed mechanism is based on the androgen’s receptor induction of cyclin D1 expression, macrophage M2 polarization and direct effect on lung cancer growth [24]. Preclinical studies using lung cancer cell lines showed strong expression of androgen receptors, while treatment of a lung adenocarcinoma cell line with androgen led to a down-regulation of genes involved in cellular respiration [16,25]. Additionally, androgen receptor expression has been characterized in archival samples of human lung cancer tissue, and its expression appeared to counteract the negative impact of high Ki67 expression in these patients [26,27]. We also observed expression of the androgen receptor in archival tissue samples of lung cancer, with higher expression noted in adenocarcinoma compared to squamous tumors (Owonikoko et al., unpublished data). Gockel et al. recently showed that potent degradation of the androgen receptor in lung cancer cells is feasible through enzalutamide-based proteolysis-targeting chimeras [28].

Prior retrospective studies of clinical data also showed a 23% reduction in incidence rate of lung cancer among patients with prior diagnosis of prostate cancer when compared to the general population, but this study was not designed to analyze for the impact of specific treatment interventions such as ADT [18]. We have, in this study, expanded the findings from this prior study by showing that ADT for prostate cancer led to an improved outcome for lung cancer. Surprisingly, this improved outcome was limited to Caucasian patients on subset analysis. Our inability to demonstrate similar benefits of ADT in Black patients could be due to the smaller number of Black patients in our analytic dataset. However, it could also suggest that ADT is less effective and potentially even detrimental in Black patients compared to Caucasian patients. Previous studies showed that a significant delay exists in the initiation of ADT for Black and Hispanic prostate cancer patients, and these ethnic groups that align with those of low socioeconomic status were, in general, less likely to receive ADT instead of surgical castration [29,30]. A retrospective study of the safety of a short course of neoadjuvant ADT in low-risk or favorable intermediate-risk prostate cancer also showed an increased risk of death in Black patients (adjusted HR of death: 1.77; 95% CI: 1.06–2.94; *p* = 0.028) [31].

Our interesting findings should be interpreted with caution due to the limitations of a retrospective study that we were unable to fully control for in the study. Current treatment for advanced stage lung cancer is strongly guided by the molecular characteristics of the tumor. EGFR mutant lung cancer is the most relevant subset of lung cancer with regards to the biology of steroid hormone signaling and interaction with lung cancer specific therapy. However, the SEER–Medicare database does not include molecular information of the patients. This limitation makes it impossible for us to fully associate our findings with the most relevant subset of lung cancer. Another limitation is the incomplete information about the various interventions employed to antagonize androgen receptor signaling in prostate cancer, such as the dose, duration and frequency of ADT. We did not have data about oral androgen receptor antagonists, which are rapidly becoming part of the standard of care for patients in earlier stage prostate cancer [32,33,34,35,36,37]. It is, therefore, possible that some of the patients classified as lacking ADT exposure could have been misclassified. This is unlikely to be the case for most of the patients, since these alternative interventions are employed along with or as salvage after failure of the parenterally administered ADT. Moreover, any misclassification would still not negate our findings and, in fact, it would suggest that the noted observed differences would have been stronger if those patients had not been included in the ADT non-exposed group. Further, the multivariate analysis did not account for all potential confounding factors such as comorbid conditions and other medications that patients were taking. While most of the study period (1988–2005) predates the FDA approval of docetaxel for hormone-refractory prostate cancer in 2004, the multivariate analysis also did not account for additional cancer-directed systemic therapies that patients could have been receiving. Despite these important limitations, our findings still provide strong testable hypotheses which can be validated or disproved in future prospective studies.

In conclusion, our study suggests a survival benefit with ADT in a subgroup of lung cancer patients who had a prior diagnosis of prostate cancer requiring ADT therapy. These real-world data support the hypothesis implicating androgen receptor signaling as a potential therapeutic target in lung cancer. External validation using institutional patient-level data will help confirm the findings. Further, the findings open the door to future preclinical and clinical studies of ADT to improve patient outcome.

## Figures and Tables

**Table 1:** Overall summary of patients and tumor characteristics.

Variable	Level	N = 367,750	%
Age at Diagnosis of Lung Cancer	<65 years	56,847	15.5
	≥65 years	310,903	84.5
Gender	Male	210,256	57.2
	Female	157,494	42.8
Race	Asian	8245	2.3
	Black	33,882	9.3
	Hispanic	3540	1.0
	White	307,343	84.2
	Other	12,032	3.3
	Missing	2708	-
Lung Cancer Stage	I	44,481	19.4
	II	7339	3.2
	III	77,533	33.8
	IV	100,298	43.7
	Missing	138,099	-
Prostate Cancer Stage	I	175	5.6
	II	1267	40.5
	III	838	26.8
	IV	852	27.2
	Missing	10,946	-
Subgroup of Lung Cancer Patients	Prostate Cancer followed by Lung Cancer (P-L)	11,061	3.0
	Lung Cancer followed by Prostate Cancer (L-P)	3017	0.8
	Lung Cancer without Prostate Cancer (L)	353,672	96.2
Medicare Status Qualifying Event	Not Enrolled	94	-
	Age	333,101	90.6
	Age with ESRD	1292	0.4
	Disabled	32,715	8.9
	Disabled with ESRD	229	0.1
	ESRD only	319	0.1

ESRD: End-Stage Renal Disease.

**Table 2:** Comparison of patient and tumor characteristics between various subgroups of lung cancer patients, P-L, L and L-P.

Covariate	Level	P-L N = 11,061	L-P N = 3017	L N = 353,672	Parametric *p*-Value *
Age at Diagnosis of Lung Cancer	<65 years	571 (5.16)	817 (27.08)	55,459 (15.68)	<0.001
	≥65 years	10,490 (94.84)	2200 (72.92)	298,213 (84.32)	
Age at Diagnosis of Prostate Cancer	<65 years	1971 (17.82)	437 (14.48)		<0.001
	≥65 years	9090 (82.18)	2580 (85.52)		
Race	White	8879 (80.78)	2380 (79.31)	296,084 (84.34)	<0.001
	Black	1502 (13.67)	461 (15.36)	31,919 (9.09)	
	Other	241 (2.19)	76 (2.53)	11,715 (3.34)	
	Asian	242 (2.2)	60 (2)	7943 (2.26)	
	Hispanic	127 (1.16)	24 (0.8)	3389 (0.97)	
Histologic Subtypes of Lung Cancer	NSCLC	8582 (77.6)	2656 (88.0)	263,758 (74.6)	
	SCLC	250 (2.3)	36 (1.2)	11,821 (3.3)	
	Carcinoma In Situ and Other Histologies	2229 (20.2)	325 (10.8)	78,093 (22.1)	
Distribution by Stage of Lung Cancer at Diagnosis	I	1382 (19.8)	824 (54.1)		
	II	199 (2.9)	99 (6.5)		
	III	2386 (34.2)	323 (21.2)		
	IV	3009 (43.1)	277 (18.2)		

L: Lung Cancer without Prostate Cancer; L-P: Lung Cancer followed by Prostate Cancer; P-L: Prostate Cancer followed by Lung Cancer; NSCLC: Non-Small Cell Lung Cancer; SCLC: Small Cell Lung Cancer. * *p* < 0.001

**Table 3:** Time latency in months between preceding prostate cancer diagnosis and subsequent lung cancer diagnosis according to clinical and tumor characteristics.

Latency from Prostate Cancer to Lung Cancer Diagnosis in the P-L Subgroup							
Variable		N	Mean	Median	ANOVA	Kruskal-Wallis	
					*p*-Value	*p*-Value	
Stage at Diagnosis of Lung Cancer	I	1382	55.19	42.00	<0.001	<0.001	
	II	199	52.59	40.00			
	III	2386	62.44	48.00			
	IV	3009	59.57	46.00			
Stage of Prostate Cancer	I	133	61.29	47.00		<0.001	
	II	822	45.82	32.00			
	III	690	69.39	63.00			
	IV	537	41.90	30.00			
Androgen Deprivation Therapy	No	7887	60.95	47.00	<0.001		
	Yes	2358	53.75	40.00			
Androgen Deprivation Therapy (Caucasian patients)	No	6775	62.17	48.00	<0.001		
	Yes	1992	54.52	41.00			
Androgen Deprivation Therapy (Black patients)	No	1112	53.54	41.00	0.166		
	Yes	366	49.54	33.00			
Latency from lung cancer to prostate cancer diagnosis in the L-P subgroup							
Variable		N	Mean	Median		*p*-value	
Stage at Diagnosis of Lung Cancer	I	824	46.96	34.00		<0.001	
	II	99	34.99	26.00			
	III	323	27.90	15.00			
	IV	277	13.27	1.00			
Stage of Prostate Cancer	I	28	54.93	38.00		0.004	
	II	348	59.64	38.00			
	III	89	55.73	42.00			
	IV	228	51.13	22.00			

L-P: Lung Cancer followed by Prostate Cancer; P-L: Prostate Cancer followed by Lung Cancer.

## Data Availability

The data presented in this study are available in this article (and supplementary material).

## Supplementary Materials

The following supporting information can be downloaded at: https://www.mdpi.com/article/10.3390/cancers14133206/s1, Figure S1: Kaplan–Meier plot for overall survival in the patients with concurrent lung and prostate cancer (P-L, L-P) as well male and female patients with only lung cancer diagnosis, Figure S2: Kaplan–Meier plot for overall survival in the L-P subgroup showing survival by stage in Black (left) and Caucasian (right) lung cancer patients, Figure S3: Kaplan–Meier plot for overall survival comparing patients treated with and without ADT in Black (left) and Caucasians (right) patients.
