# Supplementary material for: Concurrent Androgen Deprivation Therapy for Prostate Cancer Improves Survival for Synchronous or Metachronous Non-Small Cell Lung Cancer: A SEER–Medicare Database Analysis
Source: Cancers (Basel). 2022 Jun 30;14(13):3206. doi: 10.3390/cancers14133206 (PMC9265064; doi:10.3390/cancers14133206)
Supplement: Supplementary file 1 [file cancers-14-03206-s001.zip › cancers-1778201-supplementary.pdf]

Figure S1: Kaplan-Meier plot for overall survival (OS) in the patients with concurrent lung and prostate cancer (PL, LP) as well male and female patients with only lung cancer diagnosis

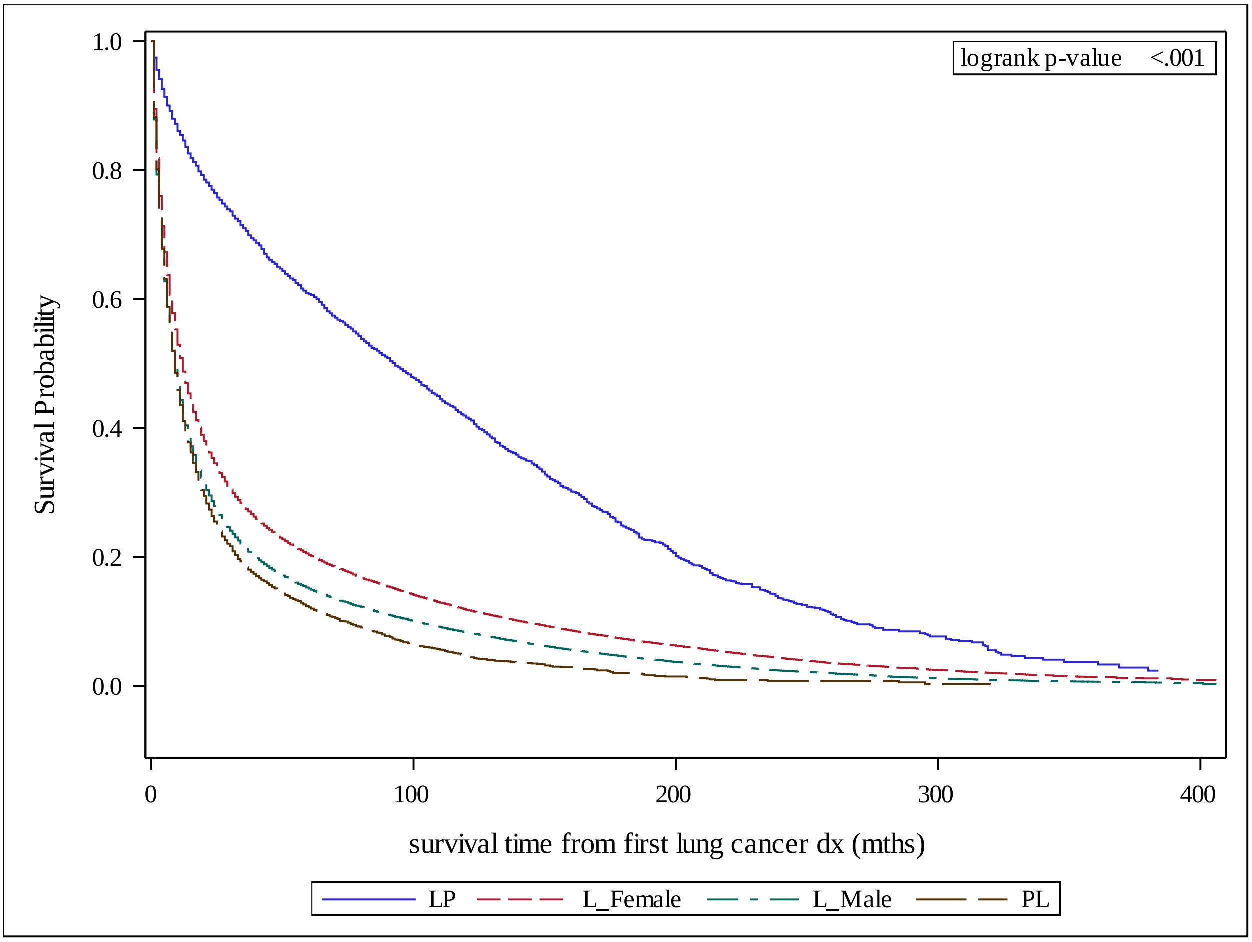

**Subgroups**	**N**	**Median OS (95% CI)**	**HR (95%CI)**	**1-Year OS (95% CI)**
L_Female	143350	12 (NA, NA)	-	48.8% (48.5%, 49.0%)
LP	2930	93 (87, 99)	0.55 (0.52-0.58); <.001	84.6% (83.2%, 85.9%)
L_Male	178728	9 (NA, NA)	1.19 (1.18-1.20); <.001	42.3% (42.0%, 42.5%)
PL	10263	9 (NA, NA)	1.14 (1.11-1.17); <.001	41.1% (40.2%, 42.1%)

HR: Hazard Ratio; L: Lung Cancer without Prostate Cancer; LP: Lung Cancer followed by Prostate Cancer; OS: Overall Survival; PL: Prostate Cancer followed by Lung Cancer.

Figure S2: Kaplan-Meier plot for overall survival in the LP subgroup showing survival by stage in Blacks (left) and Caucasian (right) lung cancer patients

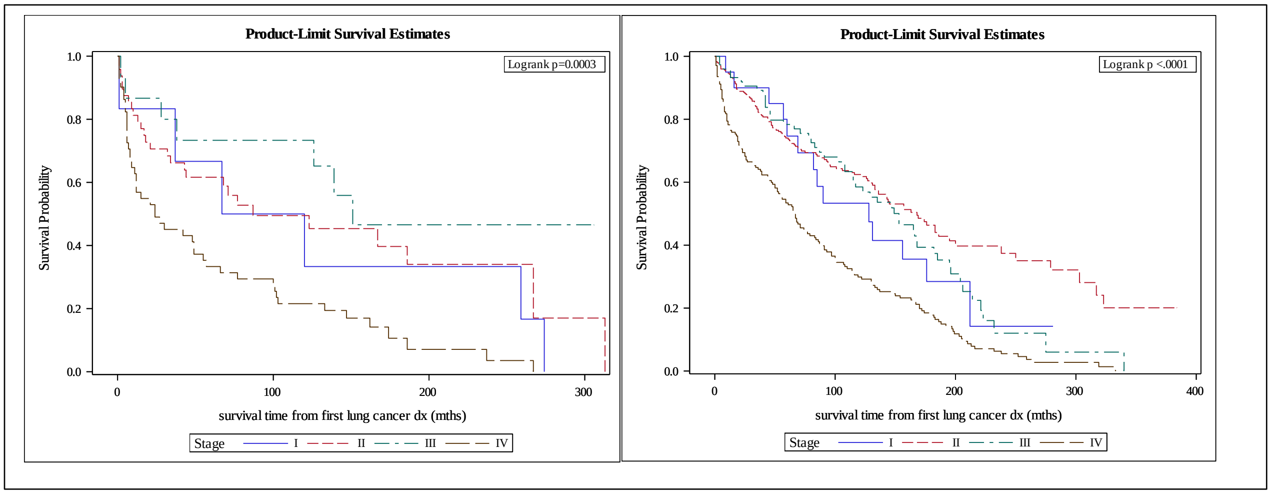

	Blacks			Caucasians		
**Stage**	**N**	**Median OS (95% CI)**	**1-year OS (95% CI)**	**N**	**Median OS (95% CI)**	**1-year OS (95% CI)**
I	6	93.5 (1, 274)	83.3% (27.3%, 97.5%)	20	128 (60, 212)	95.0% (69.5%, 99.3%)
II	48	87 (43, 267)	81.2% (67.1%, 89.8%)	298	168 (136, 195)	94.3% (91.0%, 96.4%)
III	15	151 (28, NA)	86.7% (56.4%, 96.5%)	74	153 (115, 182)	95.9% (88.0%, 98.7%)
IV	51	24 (9, 55)	56.9% (42.2%, 69.1%)	170	66 (52, 83)	78.2% (71.2%, 83.7%)

OS: Overall Survival, LP: Lung Cancer followed by Prostate Cancer.

Figure S3: Kaplan-Meier plot for overall survival comparing patients treated with and without ADT in Blacks (left) and Caucasians (right)

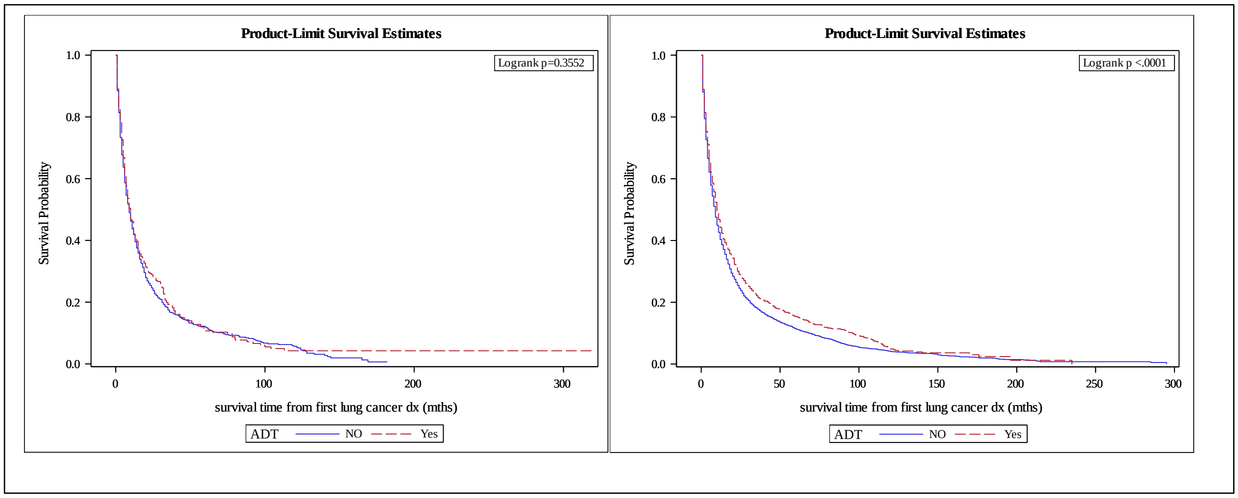

ADT (Blacks)	N	Median OS (95% CI)	1-year OS (95% CI)	2-year OS (95% CI)	5-year OS (95% CI)
No	1052	9 (8, 10)	41.8% (38.8%, 44.8%)	24.6% (22.0%, 27.3%)	11.9% (9.9%, 14.1%)
Yes	347	10 (8, 12)	42.1% (36.8%, 47.2%)	28.9% (24.2%, 33.8%)	10.7% (7.5%, 14.6%)

ADT (Caucasian)	N	Median OS (95% CI)	1-year OS (95% CI)	2-year OS (95% CI)	5-year OS (95% CI)
No	6341	9 (8, 9)	40.3% (39.1%, 41.5%)	24.6% (23.5%, 25.6%)	11.3% (10.5%, 12.2%)
Yes	1896	10 (10, 11)	44.2% (41.9%, 46.4%)	29.0% (26.9%, 31.1%)	15.4% (13.6%, 17.2%)

ADT: Androgen Deprivation Therapy; OS: Overall Survival.
